# Technical Note: MRI-guided breast biopsy - our preliminary experience

**DOI:** 10.4103/0971-3026.69362

**Published:** 2010-08

**Authors:** Sangeeta Taneja, Amarnath Jena, Kapil Kumar, Anurag Mehta

**Affiliations:** Departments of MRI, Surgical Oncology, Pathology, Rajiv Gandhi Cancer Institute & Research Centre, Sector-5, Rohini, New Delhi - 110 085, India

**Keywords:** MR-guided breast biopsy, MR mammography, wire localization

## Abstract

The diagnostic potential of breast MRI can be fully utilized only when it is possible to biopsy lesions detected on MRI, especially when they are not visible on mammography or USG. We would like to describe our experience with MRI-guided wire localization and biopsy.

## Introduction

Dynamic contrast-enhanced MRI of the breast (DCE-MRI) is a sensitive modality to pick up small cancers in the breast.[1] Nevertheless, normal parenchyma and many benign lesions may enhance and may mimic malignancy, both morphologically and on dynamic contrast characteristics. This reduces the specificity of breast MRI. Often, these lesions are seen only on MRI and are occult on mammography, USG and clinical examination. In women at high risk for breast cancer, MRI detects cancer not seen on mammography or clinical examination in 2%- 7% of women.[2] According to the American Cancer Society guidelines for breast screening with MRI as an adjunct to mammography, the ability to perform MRI-guided biopsy is absolutely essential when offering screening MRI, as many cancers (particularly early cancers) will be identified only on MRI.[3] Though MRI-guided breast biopsy has been performed in many countries in the world for many years now,[4] it has not been popular in India. We would like to share our initial experience with MRI-guided breast localizations and biopsies.

## Technical note

### Equipment and devices

**Figure 1 (a-c):**
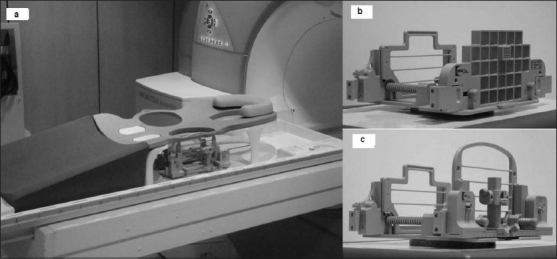
Biopsy coil device. Photographs show a four-channel breast biopsy coil with positioning device (a), a grid-positioning device (b) and a pillar-and-post positioning device (c)

The MRI-guided breast biopsies were performed on a 1.5-T scanner (AVANTO, SIEMENS, Erlangan, Germany) with a dedicated 4-channel breast biopsy coil and positioning device (320PA, NORAS, Germany) which can either be a grid or pillar and post positioning device [Figures 1a–c]. The positioning device has medial and lateral compression plates for moderate compression, a needle guide for placing the fiducial marker (filled with 1:100 solution of gadolinium and saline) and a needle sleeve. Coordinate calculation was done using the scanner software (Syngo, SIEMENS, Germany).

**Figure 2:**
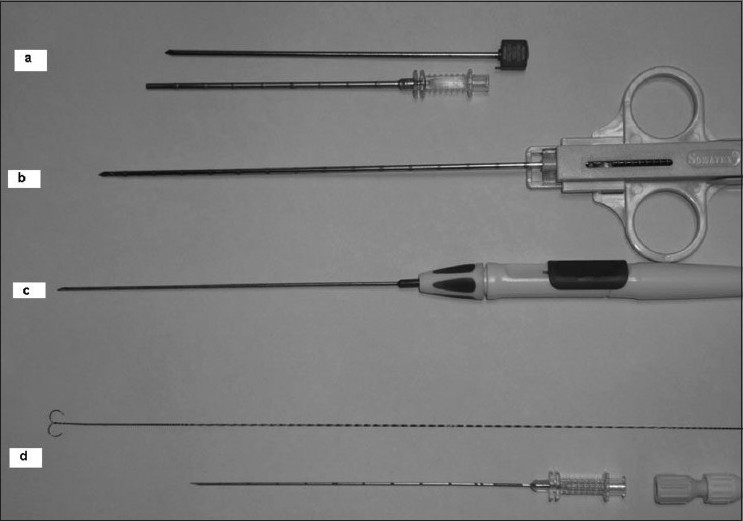
Instruments. MRI-compatible 13-G coaxial needle with trocar and cannula (a), MRI-compatible 14-G biopsy gun (b), MRI-compatible marker needle (c), MRI-compatible hook wire, cannula and locking device (d)

An MRI-compatible 13-gauge coaxial needle [Figure 2a] was used for initial puncture and for guiding the biopsy gun (MR Biopsy Handy, Somatex, Germany) [Figure 2b], which is available in three gauges, 14G, 16G and 18G and three lengths of 100 mm, 150 mm and 200 mm. MRI-safe biopsy site marker clips (TUMARK SOMATEX, GERMANY) [Figure 2c] were placed in most patients to mark the biopsy site. The marker clip is made of an approved implantable material (Nitinol) and is visible both on MRI, via its useful small imaging artifact and on mammography, to allow mammographically guided wire localization, if required. MRI-guided wire localization utilizes the same positioning device for lesion localization with an MRI-compatible needle with a hooked wire, made of a special alloy for easy and safe penetration of the solid tumor tissue (TULOC, Somatex, Germany) [Figure 2d], which can also be easily pulled back into the cannula for repositioning. It has a diameter of 0.95 mm and is available in two lengths, 90 and 120 mm. The hooks at the end of the wire fix the wire into the lesion. The wire can be fixed onto the skin with a lock which prevents any dislodgement of the wire.

### Biopsy procedure

**Figure 3 (a-d):**
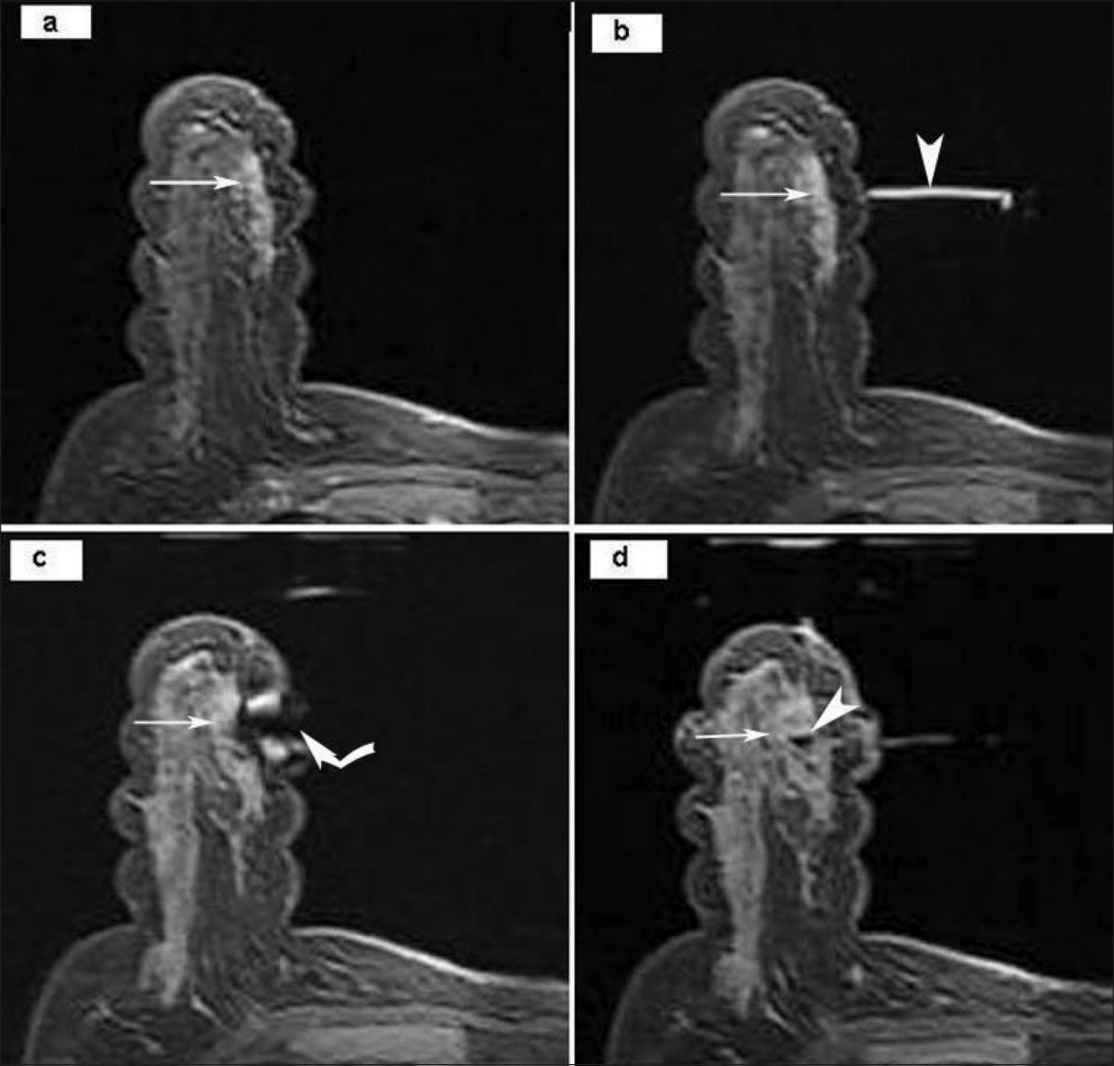
MRI-guided biopsy using axial, contrast-enhanced FLASH 3D T1W fat-suppressed images. The pre-biopsy image (a) shows an enhancing lesion (arrow) in the right breast. The fiducial marker (arrowhead in b) is seen after localization, along with the lesion (arrow). The magnetic susceptibility artifact (curved arrow) of the trocar and canula is seen (c), with the lesion (arrow) at the tip of the artifact. Following the biopsy (d), the enhancing lesion (arrow) is seen along with the small magnetic susceptibility artifact (arrowhead) due to the clip *in situ*.

Both un-enhanced and contrast-enhanced axial images were obtained to confirm the persistence of the enhancing lesion seen on a previous diagnostic MRI done at our institute or elsewhere [Figure 3a], and the locations of the fiducial marker and the lesion were recorded for inline coordinate calculation. The position of the marker was adjusted according to calculated coordinates, and the site was reconfirmed [Figure 3b]. The fiducial marker was removed and replaced by a needle guide. 2% xylocaine was used to anesthetize the skin and the breast parenchyma. A sterile 13-gauge coaxial needle was inserted to the calculated depth. With the coaxial needle in place, an axial sequence was obtained to confirm accurate placement of the needle [Figure 3c]. The inner trocar was removed, and multiple core samples of the lesion were obtained using a 14-gauge MRI-compatible biopsy gun inserted through the cannula. In most cases, an MRI-safe site marker clip was placed through the cannula after sampling, except in two patients in whom mastectomy was planned. A final axial sequence through the biopsied breast was performed to verify the location of the marker [Figure 3d].

**Figure 4:**
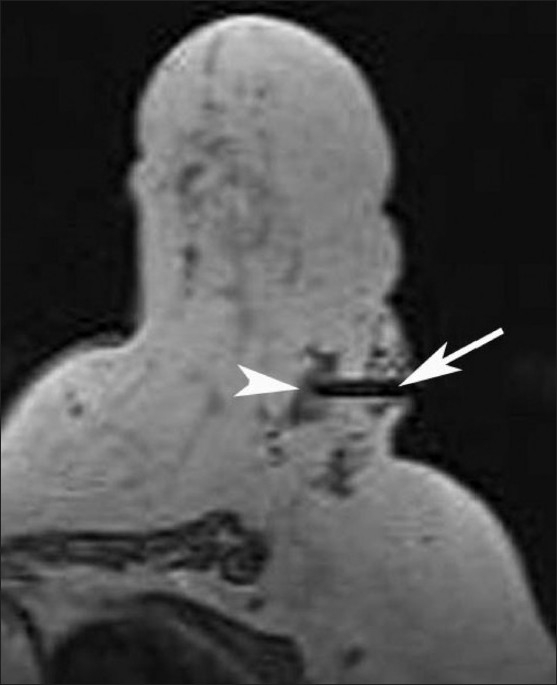
MRI-guided localization. Axial, T1W non– fat-suppressed MRI image shows a thin magnetic susceptibility artifact (arrow) due to the localization wire in place. The lesion (arrowhead) is seen near the tip

The procedure for wire placement was the same as that for lesion localization. After localizing the lesion, a cannula with a wire loaded into it was inserted to the required depth. After confirming the position on axial images, the wire was placed into the lesion and the position was verified with a repeat axial scan. The wire gives a thin linear magnetic susceptibility artifact, and the lesion can be seen near the tip of the wire [Figure 4]. It was then fixed with a lock at the skin surface to prevent dislodgement.

15 patients underwent MRI-guided interventions for MRI-only lesions. Seven patients underwent MRI-guided breast biopsy and eight patients underwent MRI-guided wire localization followed by surgery. The average lesion size was 1.2 cm (range, 0.7-1.8 cm). The average procedure time for biopsy (from the first post contrast sequence for lesion localization to the final post biopsy sequence) was 40-45 minutes and for wire localization, approximately 30 minutes.

## Results

With the pillar-and-post technique used, 1-2 cores of at least a centimeter length were obtained that were adequate for histology. Wire localization was satisfactorily done in all cases with no report of any dislodgement. 5/15 (33.3%) patients were found to have malignancy on histopathology. There were no immediate or delayed complications of the procedures.

## Discussion

Compression grid or pillar-and-post systems to immobilize the breast with the patient lying prone are the commonly used techniques, though free-hand MRI-guided interventions[5] and supine positioning[6] have has also been reported. We used the pillar and post system in all our cases, in the prone position. A lateral approach was found to be more suitable than a medial approach. None of our patients had a deep posteriorly placed lesion. However, we had difficulty in one patient who had a retroareolar lesion, since lesions placed close to the nipple cannot be captured by the biopsy grid and are difficult to target.[7] MRI-guided biopsy in implanted breasts has been reported.[8] However, we have no experience with biopsy in augmented breasts.

Fischer *et al*.,[9] in 1994 have described a technical success rate of 90% with fine needle aspiration biopsy. In a study by Kuhl and colleagues[10] in 2001, MRI-guided automated core biopsy was technically successful in 98% lesions. Vaccum-assisted biopsy has a higher technical success rate than fine needle aspiration biopsy. In an update of a multi-institutional experience, Perlet *et al*,[11] reported a success rate of 98% with MRI-guided vaccum-assisted biopsy with complications encountered in 4.7% of cases. The technical success rate of MRI-guided wire localization has been reported to be 98-100%. In a published series by Liberman *et al*.,[12] the rate of complications with MRI-guided wire localization was 3%.

Our series is small and represents our initial experience with 15 patients, 7 of whom underwent biopsy and 8, wire localization. There was no immediate or delayed complication. Adequate tissue samples could be obtained. No wire dislodgement was reported. The rate of malignancy in our limited study was 33.3%, which conforms to the previously reported findings.[13,14]

The difficulties we faced in our experience were as follows:

1. The prone position is sometimes difficult for the patient.
2. Only one breast can be biopsied at a time.
3. Deep posteriorly placed lesions and retroareolar lesions are difficult to target.

## References

[1] Morris EA (2001). Review of breast MRI: Indications and limitations. Semin Roentgenol.

[2] Morris EA, Liberman L, Ballon DJ, Robson M, Abramson AF, Heerdt A (2003). MRI of occult breast carcinoma in high risk population. AJR Am J Roentgenol.

[3] Saslow D, Boetes C, Burke W, Harms S, Leach MO, Lehman CD (2007). American Cancer Society Guidelines for Breast Screening with MRI as an adjunct to Mammography. CA Cancer J Clin.

[4] Heywang-Köbrunner SH, Huynh AT, Viehweg P, Hanke W, Requardt H, Paprosch I (1994). Prototype breast coil for MR-guided needle localization. J Comput Assist Tomogr.

[5] Daniel BL, Birdwell RL, Ikeda DM, Jeffrey SS, Black JW, Block WF (1998). Breast lesion localization: a freehand, interactive MR imaging-guided technique. Radiology.

[6] Döler W, Fischer U, Metzger I, Harder D, Grabbe E (1996). Stereotaxic add-on device for MR guided biopsy of breast lesions. Radiology.

[7] Philpotts LE, Tocino I, Lee CH (2001). Canceled stereotactic 11-gauge vacuum-assisted suction biopsy of the breast. AJR.

[8] Jackman RJ, Lamm RL (2002). Stereotactic histologic biopsy in breasts with implants. Radiology.

[9] Fischer U, Kopka L, Grabbe E (1998). Magnetic resonance guided localization and biopsy of suspicious breast lesions. Top Magn Reson Imaging.

[10] Kuhl CK, Morakkabati N, Leutner CC, Schmiedel A, Wardelmann E, Schild HH (2001). MR imaging-guided large core (14-gauge) needle biopsy of small lesions visible at breast MR imaging alone. Radiology.

[11] Perlet C, Heinig A, Prat X, Casselman J, Baath L, Sittek H (2002). Multicenter study for the evaluation of a dedicated biopsy device for MR-guided vaccum biopsy of the breast. Eur Radiol.

[12] Morris EA, Liberman L, Dershaw DD, Kaplan JB, LaTrenta LR, Abramson AF (2002). Preoperative MR imaging–guided needle localization of breast lesions. AJR Am J Roentgenol.

[13] Chen X, Lehman CD, Dee KE (2004). MRI_guided breast biopsy: Clinical experience with 14-gauge stainless steel core biopsy needle. AJR Am J Roentgenol.

[14] Han BK, Schnall MD, Orel SG, Rosen M (2008). Outcome of MRI-Guided Breast Biopsy. AJR Am J Roentgenol.

